# Bilateral Vertebral Artery Dissection in the V1 and V4 Segments Induced by Artery Anomalies and Risk Factors

**DOI:** 10.7759/cureus.85410

**Published:** 2025-06-05

**Authors:** Miharuka Yokosaki, Tomohisa Nezu, Shirou Aoki, Yu Yamazaki, Hirofumi Maruyama

**Affiliations:** 1 Department of Clinical Neuroscience and Therapeutics, Hiroshima University Graduate School of Biomedical and Health Sciences, Hiroshima, JPN

**Keywords:** bilateral vertebral artery dissection, extracranial vertebral artery dissection, intracranial vertebral artery dissection, neuro-emergency, vertebral artery anomalies

## Abstract

Bilateral vertebral artery dissection (VAD) is rare, particularly in the extracranial V1 segment. We report a case of bilateral VAD (V1 and V4 segments) in a patient with a vertebral artery anomaly, potentially triggered by the puerperium and high pillow use. A 35-year-old primiparous woman with a history of migraine developed worsening headaches after starting to use a high pillow. Computed tomography angiography revealed bilateral VAD in the V1 and V4 segments and a vertebral artery anomaly. Magnetic resonance imaging and ultrasound examinations confirmed a temporal phase difference in the intramural hematomas of both vertebral arteries. Despite having an anomalous vertebral artery for 35 years without dissection, the high pillow use during the puerperium is suspected to have triggered the dissection. Vertebral artery anomalies are present in a small percentage of the population and are not uncommon. When detected during routine health checkups, it is crucial to raise awareness of their potential risk for dissection and the importance of risk management. Furthermore, in cases of unilateral VAD, careful monitoring is warranted, as contralateral or multi-segmental dissection may be induced.

## Introduction

Vertebral artery dissection (VAD) is characterized by the formation of an intramural hematoma within the arterial wall, which can lead to a spectrum of clinical manifestations, including headache, subarachnoid hemorrhage, and cerebral infarction. VAD is recognized as one of the important etiologies of ischemic stroke in young adults. Multiple risk factors have been implicated in its pathogenesis, including migraine, pregnancy, and hereditary connective tissue disorders such as Marfan syndrome [1]. VAD typically occurs in the superior portion of the V2 or V3 segment in Caucasian patients [2] and in the V4 segment in Asian patients [3], whereas V1 segment dissection is rare. This report presents a case of bilateral VAD (involving the V1 and V4 segments) in a patient with vertebral artery anomalies. This dissection may have been triggered by overlapping risk factors, including a history of migraine, the postpartum state, and the use of a high pillow. Additionally, a time lag in the development of intramural hematomas in both vertebral arteries suggests that the bilateral dissection may have resulted from increased hemodynamic stress following unilateral dissection [4]. This report aims to highlight the importance of risk management and careful monitoring in similar cases.

## Case presentation

**Figure 1:**
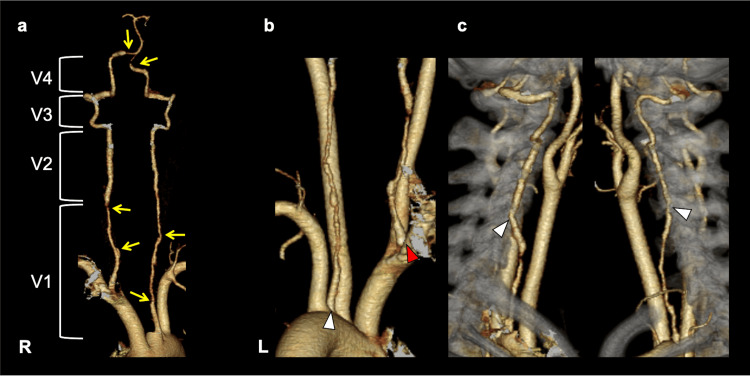
Head and neck CT angiogram (CTA) a. Irregular caliber of the bilateral V1 segments: broad, and portions of the bilateral V4 segments (yellow arrows); b. The left vertebral artery originated directly from the aortic arch (white triangle), and the right vertebral artery originated immediately after the branching of the brachiocephalic trunk (red triangle); c. Both vertebral arteries showed an anomalous high transverse foramen (C4) penetration (white triangles).

A 35-year-old, primiparous Japanese woman with a history of migraine started using a high pillow while resting after delivery to observe her child sleeping in a crib. On the eighth postpartum day, she developed left posterior neck pain upon waking. On the eleventh postpartum day, while resting with her head elevated on pillows, she suddenly developed severe pain on the right side of her neck and head, prompting an emergency call. Upon arrival at the emergency department, she was alert, with a blood pressure of 169/103 mmHg and a heart rate of 66 bpm. She complained of a right temporal headache but showed no signs of nausea or neurological deficits. As a life-threatening subarachnoid hemorrhage was suspected, an urgent noncontrast head computed tomography (CT) was performed, but no intracranial hemorrhage was observed. However, a head and neck CT angiogram (CTA) revealed an irregular caliber of the bilateral vertebral arteries (VAs) (V1 segments broad lesions and portions of the bilateral V4 segments) and bilateral VA anomalies: the left VA (LVA) originated directly from the aortic arch, and the right VA (RVA) originated immediately after the branching of the brachiocephalic trunk (Figures 1a, 1b). Both VAs showed an anomalous high transverse foramen (C4) penetration (Figure 1c).

**Figure 2:**
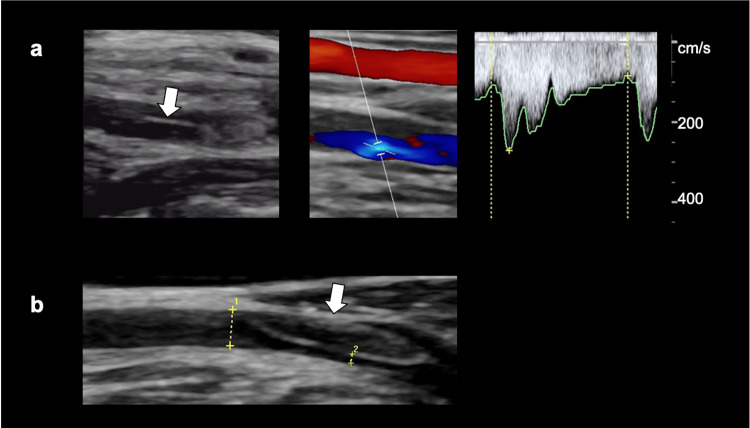
Carotid artery ultrasound a. The right vertebral artery showed a flap in the proximal V1 segment (white arrow) and a mosaic flow pattern with stenosis in the distal V1 segment (peak systolic velocity: 260 cm/s); b. The left vertebral artery showed a thrombosed false lumen near its origin (white arrow).

Carotid artery ultrasound with real-time image assessment revealed that the RVA ascended parallel to the right common carotid artery, with a flap noted in the proximal V1 segment and a mosaic flow pattern with stenosis in the distal V1 segment (peak systolic velocity: 260 cm/s) (Figure 2a). The LVA also ascended parallel to the left common carotid artery and showed a thrombosed false lumen near its origin (Figure 2b). 

**Figure 3:**
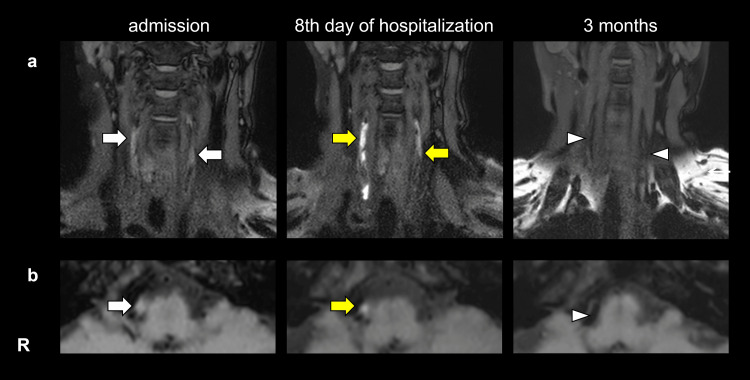
3D T1-weighted head and neck black-blood imaging (BBI) a. Changes in 3D T1-weighted imaging in the V1 segment of both vertebral arteries. On admission, 3D T1-weighted imaging revealed slight intramural hematomas in the bilateral V1 segments in both vertebral arteries (white arrows). Follow-up imaging on the eighth day of hospitalization revealed an increase in the signal intensity of the intramural hematomas (yellow arrows). Three months later, the intramural hematomas had completely resolved (white triangle). b. Changes in the BBI in the V4 segment of the right vertebral artery. On admission, BBI revealed slight intramural hematomas in the right vertebral artery (white arrow). Follow-up imaging on the eighth day of hospitalization revealed an increase in the signal intensity of the intramural hematomas (yellow arrow). Three months later, the intramural hematomas had completely resolved (white triangle).

Three-dimensional T1-weighted head and neck black-blood imaging (BBI) revealed intramural hematomas in the bilateral V1 and V4 segments. Based on these findings, a diagnosis of bilateral VAD was made, and the patient was admitted on the same day. Strict blood pressure control and conservative management (intravenous or oral analgesics and rest therapy) were implemented. Follow-up imaging revealed an increase in the signal intensity of the intramural hematomas on the eighth day of hospitalization, and as the headache improved, the patient was discharged home. Follow-up imaging three months later showed complete resolution of the hematoma (Figure 3).

Follow-up carotid ultrasound revealed persistent thrombosis of the false lumen in both VAs but progressive improvement in the true lumen, which nearly normalized after three months. The patient’s head and neck pain gradually subsided and disappeared after three months.

## Discussion

In this case, the patient presented with dissection in the bilateral V1 segments (extending from the proximal origin to the transverse foramen) and portions of the bilateral V4 segments. Vertebral arteries usually branch from the superior aspect of the subclavian artery and enter the C6 transverse foramen. However, variations in origin and transverse foramen entry are observed in a small percentage of individuals. In the current case, the LVA originated directly from the aortic arch between the left common carotid and left subclavian arteries while the RVA originated proximal to the right subclavian artery.

In a study evaluating VA anomalies in 2,287 individuals, CTA imaging revealed that the LVA branched directly from the aortic arch in 4.1% of cases, while the RVA originated from the proximal portion of the right subclavian artery in 3.1% of cases [5]. These anatomical variations are associated with a higher entry into the transverse foramen [6], resulting in a more anterior and longer ascending course in extracranial VAs. It is postulated that a VA origin closer to the heart exposes it to stronger pulsatile blood flow, and the increased length of the cervical VA (V1) before it enters the transverse foramen may lead to increased shear stress, predisposing it to dissection [7]. In addition, the postpartum period, a history of migraine headaches, and the use of high pillows have each been identified as potential risk factors [8-10]. Although no case reports have documented a patient presenting with all of these factors simultaneously, it is conceivable that the combination of multiple risk factors may have contributed to increased arterial wall vulnerability, ultimately resulting in vertebral artery rupture in this case.

Two mechanisms have been reported for the development of bilateral VAD: the propagation of unilateral dissection to the contralateral side through VA union [11] and increased blood flow load on the contralateral side due to decreased blood flow from the unilateral dissection, triggering contralateral dissection [4]. In this case, the symptoms initially presented as left cervical pain, followed by right posterior neck pain. CTA imaging revealed no irregular caliber in the V2 and V3 segments, and T1-BBI showed no high signal intensity. Carotid ultrasound suggested a slight difference in onset timing rather than the simultaneous dissection of both V1 segments. Given the aforementioned findings, the mechanism of bilateral VAD can be described as follows. First, dissection of the LVA (V1) occurs due to anomalies, leading to a decrease in blood flow in the LVA. Consequently, the hemodynamic load on the RVA increases, potentially triggering dissection of the RVA. The occurrence of unilateral VAD, particularly in patients with VA anomalies, may increase the risk of subsequent contralateral or multi-segmental dissection. Therefore, careful follow-up is needed.

## Conclusions

We reported this case of bilateral VAD in the V1 and V4 segments in a patient with bilateral VA anomalies, potentially triggered by migraine, a postpartum state, and the use of a high pillow. Vertebral artery anomalies may increase the risk of developing VAD due to altered hemodynamics and increased mechanical stress. Vertebral artery anomalies are present in a small percentage of the population and are not uncommon. Also, arterial dissection can lead to serious complications, including subarachnoid hemorrhage and cerebral infarction. When detected during routine health checkups, it is crucial to raise awareness of their potential risk for dissection and the importance of risk management. Furthermore, in cases of unilateral VAD, careful monitoring is warranted, as contralateral or multi-segmental dissection may be induced.
